# Quantifying Spatiotemporal Changes in Human Activities Induced by COVID-19 Pandemic Using Daily Nighttime Light Data

**DOI:** 10.1109/JSTARS.2021.3060038

**Published:** 2021-02-18

**Authors:** Ting Lan, Guofan Shao, Lina Tang, Zhibang Xu, Wei Zhu, Lingyu Liu

**Affiliations:** 1 Key Laboratory of Urban Environment and HealthInstitute of Urban EnvironmentChinese Academy of Sciences 85406 Xiamen 361021 China; 2 University of Chinese Academy of Sciences 556346 Beijing 100049 China; 3 Department of Forestry and Natural ResourcesPurdue University 311308 West Lafayette IN 47907 USA; 4 Key Laboratory of Urban Environment and HealthInstitute of Urban EnvironmentChinese Academy of Sciences 85406 Xiamen 361021 China; 5 School of Resource and Environmental SciencesWuhan University 12390 Wuhan 430079 China

**Keywords:** China, COVID-19, National Polar-Orbiting Partnership-Visible Infrared Imaging Radiometer Suite (NPP-VIIRS) day/night band (DNB) daily data, nighttime light (NTL)

## Abstract

The COVID-19 pandemic caused drastic changes in human activities and nighttime light (NTL) at various scales, providing a unique opportunity for exploring the pattern of the extreme responses of human community. This study used daily NTL data to examine the spatial variations and temporal dynamics of human activities under the influence of COVID-19, taking Chinese mainland as the study area. The results suggest that the change in the intensity of NTL is not correlated to the number of confirmed cases, but reflects the changes in human activities and the intensity of epidemic prevention and control measures within a region. During the outbreak period, the major provincial capitals and urban agglomerations were affected by COVID-19 more than smaller cities. During the recovery, different regions showed different recovery processes. The cities in West and Northeast China recovered steadily while the recovery in coastal cities showed relatively greater fluctuations due to an increase in imported cases. Wuhan, the most seriously affected city in China, did not recover until the end of March. Nevertheless, as of 31 March, the overall NTL across China had recovered to an 89.5% level of the same period in the previous year. The high consistency between the big data of travel intensity and NTL further proved the validity of the results of this study. These findings imply that daily NTL data are effective for rapidly monitoring the dynamic changes in human activities, and can help evaluate the effects of control measures on human activities during major public health events.

## I. Introduction

With the development of human society, lights have gradually been popularized in human settlements, and high light intensities are now emitted at night. Nighttime light (NTL), which can be detected by remote sensing technology, is closely related to human activities [1]. NTL data can reflect the lights of cities, towns, and other sources (e.g., fishing boats, natural gas burning, and forest fires) at night. Therefore, such data are widely used in many fields. NTL data are effective for assessing long-term socioeconomic parameters such as gross domestic product (GDP) [2], [3], human population [4], [5], electrical power consumption [6], [7], greenhouse gas emissions [8], and poverty [9]; for quantifying urbanization [10]–[15] and urban sprawl [16]–[18]; for monitoring the impacts of unexpected events, such as war [19]–[21], natural disasters (e.g., typhoons, earthquakes, and floods) [22], [23], large-scale power outages [24]; for tracing fishery [25]; and for analyzing environmental effects [26], [27] and assessing issues such as light pollution [28], [29], animals’ living habits [30]–[32], and human physiological and psychological health [33]–[38], etc.

Although NTL data have been widely used in studies of the environment and health, due to the unclear dynamic relationship between epidemics and light, few studies have investigated the dynamic relationship between NTL intensity and human activities under the influence of an epidemic [23]. Previous studies have shown that dramatic changes in light occur before and after wars and natural disasters, and the analysis of these light changes can reflect the severity of the event and its impact on human society [20], [23]. Epidemics, wars, and natural disasters all have a great impact on human society; however, the impact of epidemics is different from those of the latter two. First, the duration is different. The duration of natural disasters (e.g., typhoons, earthquakes, and floods) is short, generally lasting for a few hours or days, while epidemics may last for months or longer. Second, the forms of expression are different. Wars and natural disasters are tangible, whereas an epidemic can be considered as a “war without smoke.” Furthermore, the occurrence of wars and natural disasters leads to changes in light by causing serious damage to human settlements, commercial districts, industrial districts, etc.; however, epidemics do not destroy these infrastructures, rather causing changes in light mainly by affecting human activities [38].

In December 2019, COVID-19 broke out in China [39], [40], and the outbreak was active at the time of the Spring Festival transport season in 2020. Spring Festival transport is a mass population migration phenomenon unique to China that involves large-scale high-pressure transportation before and after the Lunar New Year. Population migration during the Chinese Spring Festival in 2019 totaled 2.98 billion according to the Ministry of Transport of the People's Republic of China.^1^^1^[Online]. Available: http://www.mot.gov.cn/jiaotongyaowen/201903/t20190302_3170940.html. Under the double pressure of the Spring Festival transportation and the COVID-19 outbreak, epidemic prevention and control were dire. In order to prevent the rapid spread of COVID-19 due to the mass migration of people, on 23 January 2020, the city of Wuhan and various provinces in China launched a first-level public health emergency response and suspended social and economic activities. The “freezing” of human flow rapidly reduced the number of people infected with COVID-19, and the outbreak reached a turning point on 18 February. Since 10 February, China's social economy has gradually recovered and human migration has increased. By the end of March 2020, China had 82 601 confirmed cases of COVID-19.

During the COVID-19 outbreak, in less than three months, human activities in China transitioned from peak to trough and then gradually recovered, which provides a rich opportunity for monitoring human activities using NTL. Besides, the emergence of COVID-19 urgently requires the provision of technology to rapidly assess the spatial impact of the epidemic. Therefore, in this study, daily NTL remote sensing data were used to quantitatively monitor the dynamic evolution of the epidemic and study the dynamic relationship between the epidemic outbreak and the radiation intensity of NTL. Additionally, this study explored scenarios for the application of NTL remote sensing technology in emergency public health events across large regions in order to provide support for the response to global public health events in the future. The scientific questions addressed in this article are: 1) what are the spatial characteristics of human activities under the influence of COVID-19? and 2) what are the characteristics of the NTL changes that were caused by changing human activities during the COVID-19 outbreak?

## II. Data and Study Area

### A. Data Source

Nowadays, many sensors are capable of detecting sources of surface light at night [41]. The most commonly used NTL are from the Defense Meteorological Satellite Program-Operational Linescan System (DMSP-OLS) and the Suomi National Polar-Orbiting Partnership-Visible Infrared Imaging Radiometer Suite (NPP-VIIRS). The former was created by the National Oceanic and Atmospheric Administration's National Centers for Environmental Information (NOAA/NCEI) and consists of a long-term digital data archive that extends from 1992 to 2013 [42]. However, DMSP-OLS data are not calibrated and have a low spatial resolution. Meanwhile, the VIIRS can detect NTL at higher spatial and radiometric resolutions than DMSP-OLS and practically eliminates three critical problems that beset the heritage satellite program—saturation, blooming, and a lack of on-board calibration [42]. Due to these advantages, in this study, NPP-VIIRS data were applied to study the influence of COVID-19 in China.

NPP-VIIRS data were obtained from the NOAA/NECI [43].^2^^2^[Online]. Available: https://ngdc.noaa.gov/eog/download.html. However, the raw NPP-VIIRS day/night band (DNB) daily data have deviation due to the influence of clouds and moonlight as well as stray light, fires, and other ephemeral lights [42]. Therefore, it is necessary to correct the daily NTL data. The NTL data used in this study include monthly composite data from December 2018 and January, February, and March 2019 and daily data from 20 January 2020 to 31 March 2020.

Population migration big data of Baidu map (PMBD) is supported by Baidu (China's largest integrated internet service company). PMBD visually represents the trajectories and features of population migration before and after the Chinese Spring Festival.^3^^3^[Online]. Available: http://qianxi.baidu.com/. PMBD are dimensionless which have been standardized by Baidu. The intensity of inner city travel data of Baidu map (IICT) is also provided by Baidu. These data are an indexed result of the ratio of the number of people traveling in a city to the city's resident population, which is a dimensionless value. This study obtained daily PMBD and IICT data from 12 January 2019 to 12 April 2019 and from 1 January 2019 to 31 March 2020.

Data for the COVID-19 epidemic from 22 January 2020 to 31 March 2020, including the number of confirmed infections (hereafter referred to as the confirmed count), number of suspected infections (suspected count), number of cured (cured count), and number of dead (dead count), were obtained from Dingxiangyuan^4^^4^[Online]. Available: https://ncov.dxy.cn/ncovh5/view/pneumonia. (a medical knowledge sharing website). The data from 22 and 23 January are measured by province and the data from 24 January to 31 March are measured by city. The city is the basic research scale used in this study. After data cleaning, eliminating duplicate and redundant data, and completing missing information, a total of 17 259 data values were obtained. The number of existing confirmed infections (the number of confirmed cases that were under treatment as of 31 March 2020) in each city was calculated by the following equation:

\begin{equation*}
{\rm{ECon}}\ = {\rm{\ Con}} - c - d\tag{1}
\end{equation*}

where ECon represents the number of existing COVID-19 infections, Con is the confirmed count, *c* is the cured count, and *d* is the dead count.

### B. Data Preprocessing

The preprocessing of the NPP-VIIRS DNB daily data mainly includes the following steps—clipping the data, setting the negative values to 0, unifying the projection coordinate system to Asia North Albers equal area conic, resampling all grids to 500 × 500 m, and most importantly, removing the influence of moonlight, cloud-cover, stray light, fires, other ephemeral lights, and noise at the edge of the swath [44].

The moon phase angle and lunar zenith angle are the critical factors affecting the lunar radiant luminance. Moonless data refer to the data for which the moon phase angle and the average moon zenith angle of each orbit data are greater than 90°. Therefore, the data from 20 January to 1 February, 16 February to 2 March, and 17 March to 1 April 2020 were not affected by moonlight. The data that were affected by moonlight were not used in this article.

The location of the noise in the NPP-VIIRS DNB daily data differs every day. Generally, the real NTL radiance of each pixel should fluctuate within a specific range without too many changes. However, when there is noise, the value of light radiation will change greatly. Therefore, 1000 points were randomly selected in the relatively dark area of the study area, and the light radiation values of these 1000 points were analyzed in time series. The results show that the values of most pixels fluctuate in the range of 0–1. In this study, the statistical method based on quartiles (SMBQ) [45] is used to identify and repair the noise from each pixel in the image. The equations are as follows:

\begin{align*}
&N > {Q_3} + 1.5\left({{Q_3} - {Q_1}} \right)\tag{2}\\
&n < {Q_1} - 1.5\left({{Q_3} - {Q_1}} \right)\tag{3}
\end{align*}

where *Q_1_* is the first quartile, which is the median of the lower half of the dataset. *Q_3_* is the third quartile, which is the median of the upper half of the dataset. *N* and *n* are outliers. *N* is corrected by the maximum value [*Q_3_*+1.5(*Q_3_-Q_1_*)] of the corresponding pixel, and *n* is corrected by the minimum value [*Q_1_* -1.5(*Q_3_-Q_1_*)] of the corresponding pixel. *N* usually represents phenomena such as scanner edge noise and fires, and *n* typically represents the pixel has cloud.

**Fig. 1.**
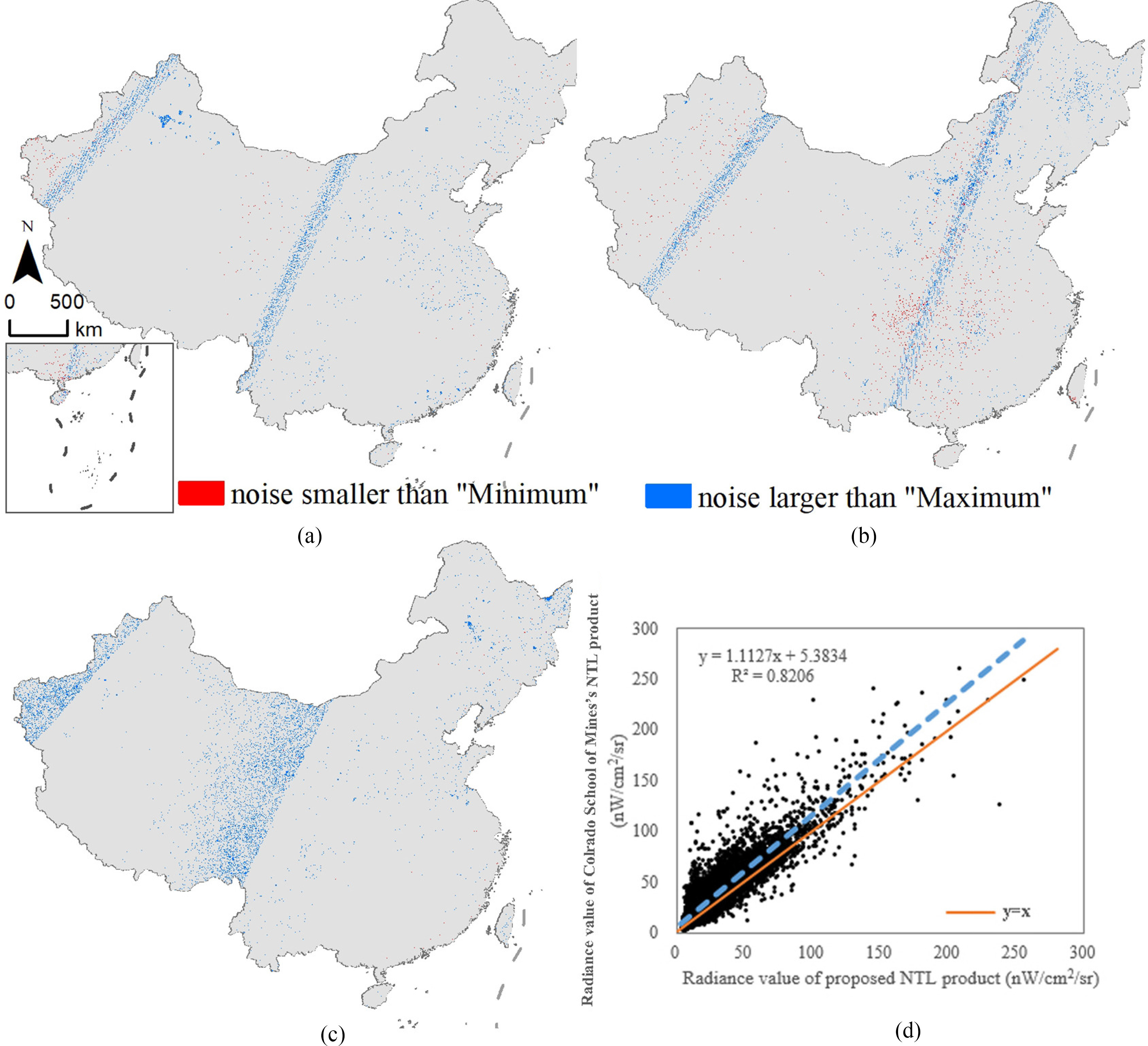
(a)–(c) Results of outlier identification. (d) Outlier repairing.

The outliers are consistent with the real situation that the number of high outliers is more than the number of low outliers, and the low outliers are mainly in Sichuan, Chongqing, and other cloudy areas [see Fig. 1(a)–(c). Then, the January NTL product downloaded from the academic sector at the Colorado School of Mines is compared with the January product produced by SMBQ to verify the accuracy of noise repairing [see Fig. 1(d)]. The former has not been filtered to screen out lights from fires, boats, and other temporal lights, and the two products have different methods for noise processing, resulting in a difference in the radiance values [see Fig. 1(d)]. The slope of the scatter plot is 1.11, R2 is 0.82, and the difference is within a reasonable error range. The results show that SMBQ can effectively repair the noise.

### C. Study Area

Due to the lack of data, this study only considered 31 provincial administrative regions in the Chinese mainland but did not include Hong Kong, Macao, and Taiwan. According to the data from the Chinese National Bureau of Statistics, in 2019, the permanent population of these 31 provinces was about 1403.85 million, accounting for 97.79% of the country's total population. Besides, there are 367 cities in these 31 provinces, each with a different population, different population flow characteristics, and different epidemic levels.

## III. Methods

**Fig. 2.**
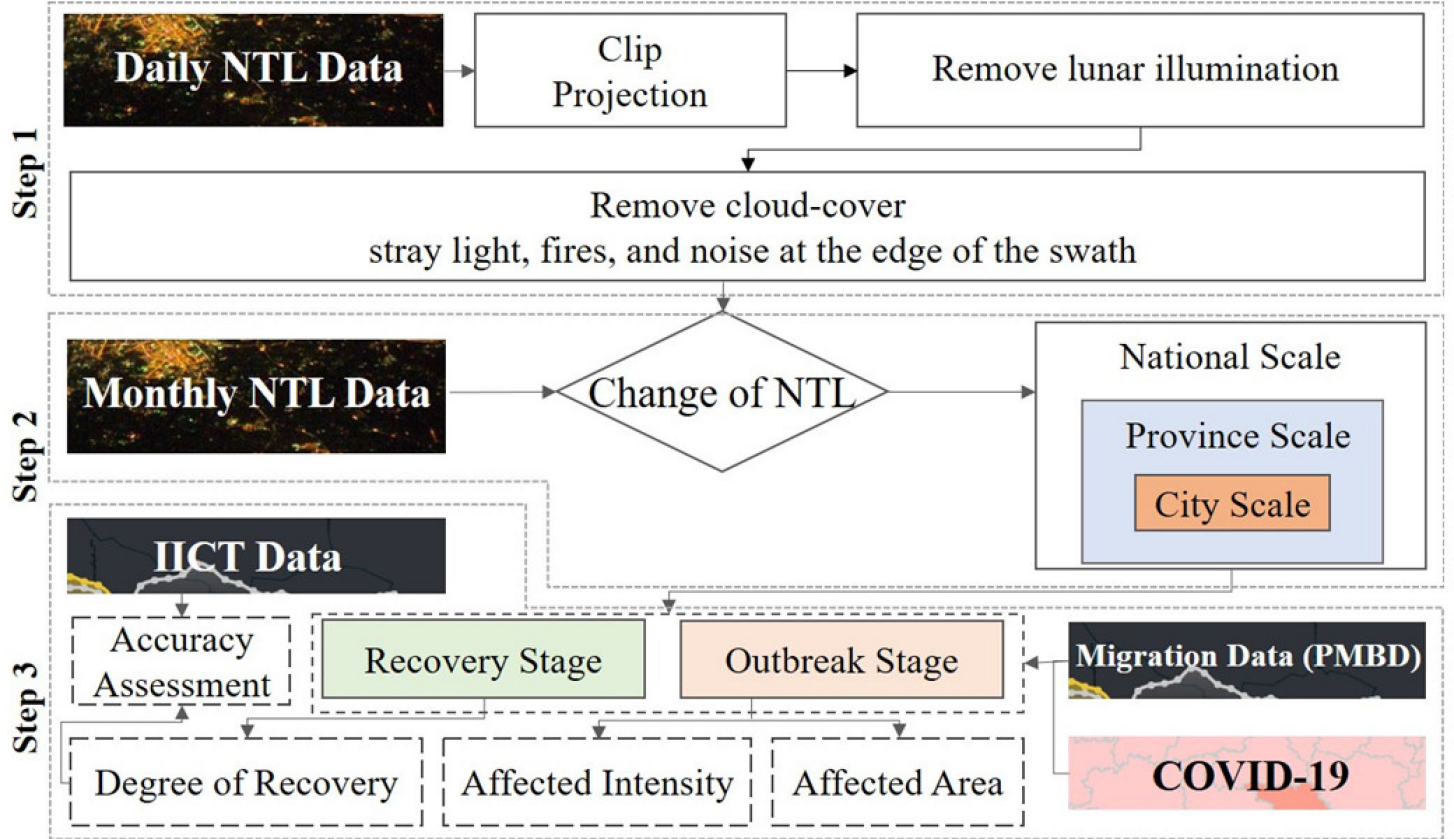
Workflow of this study.

First, the VIIRS DNB daily data for 2020 were preprocessed, removing the lunar illumination. Additionally, based on SMBQ, the outliers of VIIRS DNB daily data are repaired. Then, according to the differences between the daily NTL data in 2020 and the monthly NTL data on the same day in the 2019 Chinese lunar calendar, the spatial differences and dynamic processes of human activities under the influence of COVID-19 were analyzed at the national scale, provincial scale, and urban scale. According to the PMBD data and the existing COVID-19 infections, the time interval of the study was divided into two periods—the outbreak period (S1: 23 January–18 February) and the recovery period (S2: 19 February–31 March). Finally, the spatial characteristics of changes in urban NTL during the epidemic, the impact of the epidemic in each city, and the spatiotemporal characteristics of the cities’ recovery degree were analyzed. The technical workflow is shown in Fig. 2.

### A. Affected Area

The most common way to monitor changes in NTL is to compare NTL values at different times, including at the pixel level [23], [24], [46], and the regional level [47]. In 2020, the NTL in China was affected by the Spring Festival and the COVID-19 epidemic, while in 2019, at the same period of the Chinese lunar calendar, the NTL was only affected by the Spring Festival. Due to social and economic development, the value of NTL radiance in 2020 should be higher than that in 2019. Therefore, by only comparing the change in NTL radiance between the outbreak period of 2020 and the same period of the Chinese lunar calendar in 2019, the influence range of COVID-19 might be underestimated in some regions. Thus, it is necessary to add a development index to correct the results in this study. This index is calculated using the following formulas:

\begin{align*}
{\rm{CNT}}{{\rm{L}}_i} &= {R_i}{\rm{\ }} - {r_i} - {D_i}\tag{4}\\
{D_i} &= {d_i}{\rm{\ }} - d_i^{\prime}\tag{5}
\end{align*}

where CNTL*_i_* is the variation of the NTL radiance of grid *i*, *R_i_* are the NTL radiance of grid *i* during the outbreak in 2020, *r_i_* represents the value of NTL of grid *i* during the same period in the Chinese lunar calendar in 2019, *D_i_* is the development index of grid *i*, *d_i_* is the NTL radiance of grid *i* in December 2019, and $d_i^\prime$ is the value of NTL of grid *i* in the same period of the previous year (12 December–31 December 2018, 1 January–11 January 2019). The value of CNTL>0 shows the increase in NTL, and the value of CNTL<0 indicates the decrease in NTL. However, small changes in NTL (caused by background noise) are not caused by the epidemic, but rather by the normal fluctuation of the background light. We set different thresholds for CNTL greater than zero and less than zero until they could remove the background noise and reflect the changes of NTL in different regions. Specifically, first of all, we removed the obvious background noise. Then, according to the experience, the lights of large shopping malls are weakened, and the lights of most Tertiary Grade A hospitals are brighter during the COVID-19 epidemic; with these characteristics preserved, the threshold values were gradually adjusted, and the desired results could be obtained when the threshold values are determined to be less than -1 or more than 3.

### B. Influence Intensity

The higher the CNTL value, the more severe the impact of the epidemic. In this study, the city is the basic statistical unit for the determination of the influence intensity of the epidemic. The influence intensity of COVID-19 (SCNTL) is determined by summing the CNTL values of each city. The influence intensity of the affected area is rounded and divided into five levels based on the natural breaks method. In detail, level I represents a very high epidemic intensity, level II represents a high intensity, level III represents a medium intensity, level IV represents a low intensity, and level V represents very low intensity

\begin{equation*}
{\rm{SCNTL}}\ = \mathop \sum \limits_{i = 1}^n {\rm{CNT}}{{\rm{L}}_i}. \tag{6}
\end{equation*}

### C. City Recovery Index

It has been proved that NTL is closely related to human activity [42], [48]. In this study, NTL data from the same day in the 2019 Chinese lunar calendar are used as the control data to determine the normal NTL radiance, that is, without the impact of the epidemic. The degree of city recovery is expressed by the ratio between the NPP-VIIRS DNB daily data in the recovery period of 2020 and the same data in the same period in the Chinese lunar calendar in 2019, as calculated by the following equation:

\begin{equation*}
{\rm{RNTL}}\ = \frac{{\sum R_i^{\prime}}}{{\sum r_i^{\prime}}} \tag{7}
\end{equation*}

where RNTL represents the recovery degree of a city, $R_i^\prime$ is the NTL intensity of grid *i* in the recovery period of the epidemic in 2020, and $r_i^\prime$is the NTL intensity of grid *i* in the 2019 Chinese lunar calendar. The value of RNTL≧̸l indicates that the city has fully recovered, while the value of RNTL≦̸1 suggests that the city has not fully recovered, and the smaller the RNTL, the weaker the city's recovery.

### D. Anselin Local Moran's I Statistics

The Anselin Local Moran's I statistic was proposed by Anselin [49]. Given a set of weighted features, this statistic identifies statistically significant hot spots, cold spots, and spatial outliers, which can be used to better analyze the distribution of NTL changes in space. The formula for the Anselin Local Moran's I statistic is shown as follows:

\begin{equation*}
{{\rm{I}}_i} = \frac{{\left({n - 1} \right)\left({{x_i} - \bar{X}} \right)\mathop \sum \nolimits_{j = 1,j \ne i}^n {w_{ij}}\left({{x_i} - \bar{X}} \right)}}{{\mathop \sum \nolimits_{j = 1,j \ne i}^n {{\left({{x_i} - \bar{X}} \right)}^2}}} \tag{8}
\end{equation*}

where *x_i_* is the influence intensity for city *i*, $\bar{X}$ is the mean influence intensity for all of the studied cities, *w_ij_* is the spatial weight between cities *i* and *j* (the spatial weight was determined by inverse distance), and *n* is the total number of cities.

### E. Accuracy Assessment

To some extent, IICT reflects the correlation between the intensity of human activity and the ratio of urban recovery. Therefore, in this study, IICT is applied to perform accuracy verification. The formula for the recovery degree of IICT (*m*) is as follows:

\begin{equation*}
m\ = \frac{{{P_j}}}{{{p_j}}} \tag{9}
\end{equation*}

where *P_j_* is the IICT on day *j* in 2020 and *p_j_* is the IICT on day *j* in the 2019 Chinese lunar calendar*.*

## IV. Results

### A. Review of the Spread of COVID-19 in China

**Fig. 3.**
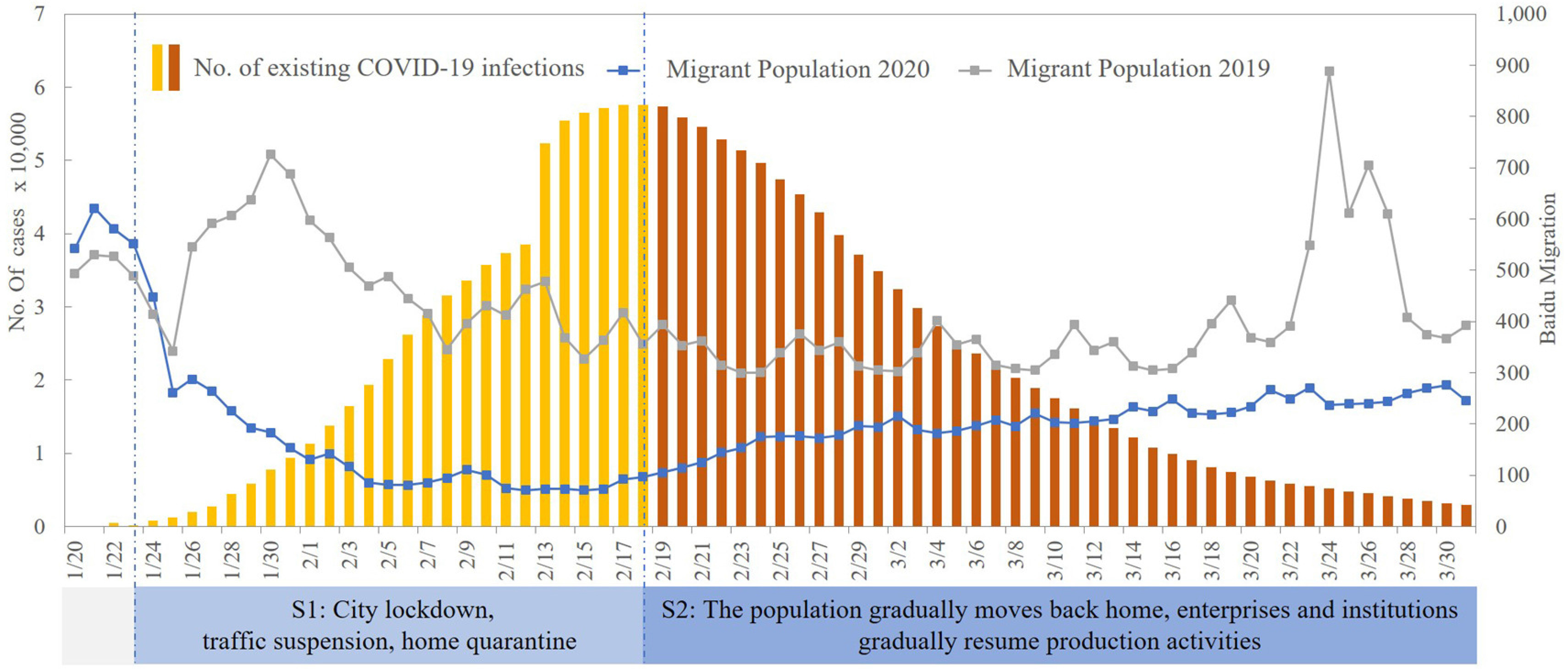
Two periods of the epidemic defined in this research.

On 23 January 2020, the Chinese government imposed a lockdown of the city of Wuhan, and by 25 January, about 30 provinces across China had activated a first-level public health emergency response. Using PMBD, it was found that the level of population migration across China fell sharply after January 23. With the implementation of epidemic prevention and control measures, the COVID-19 epidemic was gradually controlled, and a turning point occurred on February 18. By combining the characteristics of the epidemic development, the characteristics of population migration, and the implementation time of the epidemic prevention and control measures, we divided the research period into two parts—the outbreak period and the recovery period (see Fig. 3). The outbreak period is characterized by strong intervention, a significant decline in population migration, home quarantine, and the cessation of social and economic activities. The recovery period is characterized by a gradual return to work and production under the strong intervention, where the population gradually moves back to the cities where they live and work and social and economic activities gradually resume.

### B. Spatial Extent of the Epidemic's Impact

**Fig. 4.**
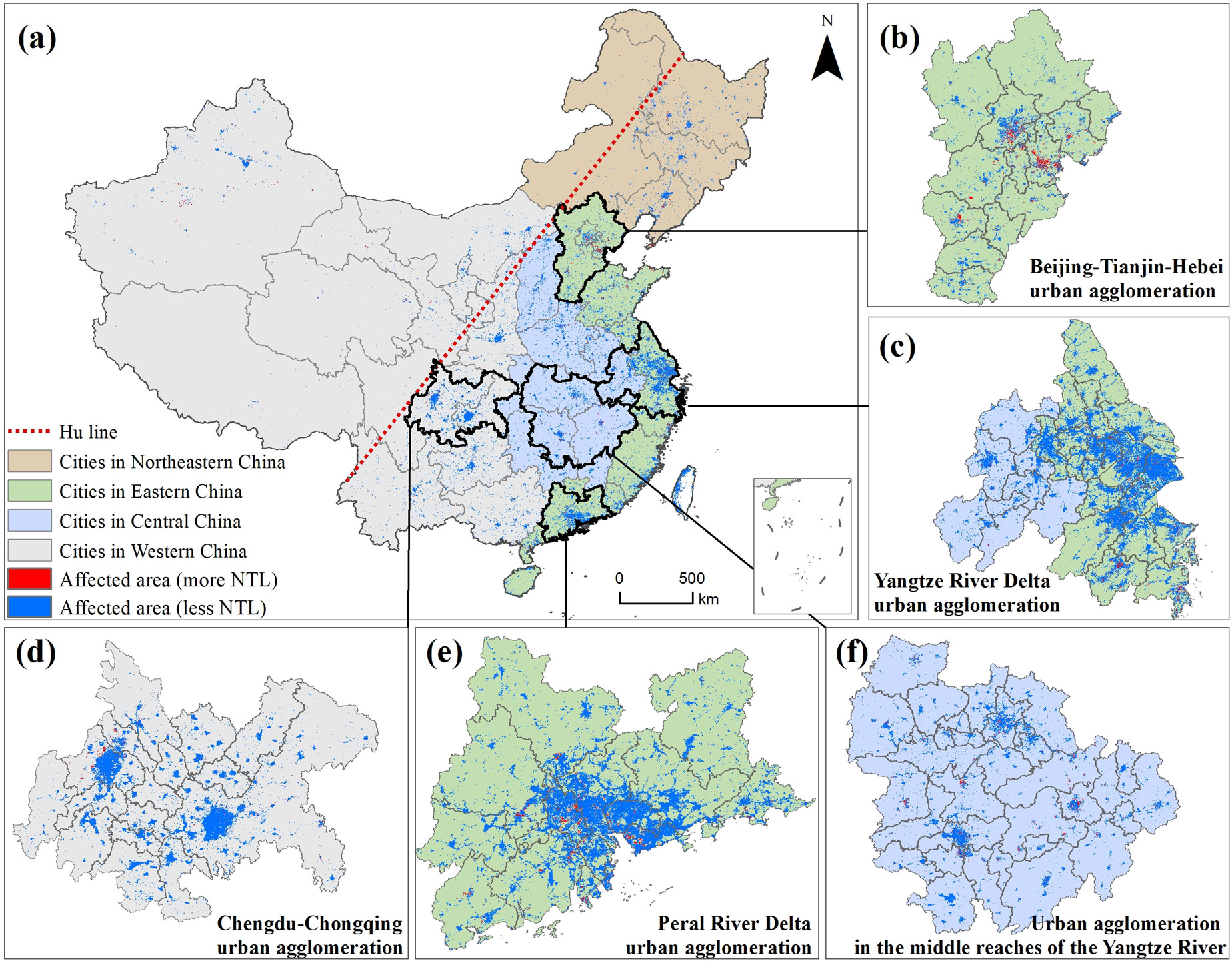
Spatial extent of the impact of COVID-19 in China.

At the outbreak period, the areas affected by COVID-19 included two types—areas where the NTL radiance became lower and areas where the NTL radiance became higher. The areas where the NTL radiance became higher are those where the intensity of human activities became higher under the influence of the epidemic. The reason why the NTL radiance became higher is the use of infrastructure for patient treatment and epidemic detection. Meanwhile, the areas where the NTL radiation value became lower are those where the intensity of human activities weakened under the influence of the epidemic. The reason why the NTL radiation value became lower is the large-scale suspension of social and economic activities (see Fig. 4).

In China, the area affected by the COVID-19 epidemic was 308 337 km^2^, of which the proportion of the area with a lower NTL radiance was 94% and the proportion of the area with a higher NTL radiance was 6% (only 0.06 times the area with a lower NTL radiance). The affected areas were mainly distributed to the east of the Hu line (a line proposed by geographer Huanyong Hu [50] that divides China into eastern and western halves, It stretches from Heihe in northeast to Tengchong in south, across China diagonally); some of the major cities in West China had large affected areas, while the affected areas in Northeast and Central China were relatively small. In Tibet and Qinghai in Western China, the NTL changes were small and the affected area was very small.

From a provincial perspective, the provinces most affected by COVID-19 were Guangdong, Jiangsu, and Zhejiang, with affected areas of 28 383, 24 859, and 20 095 km^2^, respectively. Tibet, Qinghai, and Tianjin had the smallest affected area, with 823, 1256^2^, and 2264 km^2^, respectively. The areas with significantly higher NTL radiance were Shanghai, Zhejiang, Liaoning, and Hebei. The provinces with significantly lower NTL radiance were Guangdong, Jiangsu, and Zhejiang (see Table Ⅰ).From the perspective of urban agglomerations, the NTL radiance of the five urban agglomerations all decreased in different ranges. The major urban agglomerations affected by COVID-19 are the Yangtze River Delta urban agglomerations, the Chengdu–Chongqing urban agglomerations, and the urban agglomerations of the Guangdong–Hong Kong–Macao Greater Bay Area of the Pearl River Delta, with affected areas of 47 695, 21 873, and 19 829 km^2^, respectively. The urban agglomerations in the middle reaches of the Yangtze River and Beijing–Tianjin–Hebei were slightly less affected, with affected areas of 19 561 and 15 402 km^2^, respectively. The urban agglomerations with the highest area with increased NTL radiance were the Beijing–Tianjin–Hebei urban agglomeration (2439 km^2^), the Yangtze River Delta urban agglomeration (1566 km^2^), and the middle Yangtze River urban agglomeration (1175 km^2^).

**TABLE I:** Area Affected by the COVID-19 Epidemic in Various Provinces, Regions, and Cities of China

Location	Affected area (less NTL) (km^2^)	Affected area (more NTL) (km^2^)	Affected area (km^2^)	Location	Affected area (less NTL) (km^2^)	Affected area (more NTL) (km^2^)	Affected area (km^2^)
Anhui	11 919	277	12 195	Chongqing	7984	69	8053
Beijing	2580	513	3093	Inner Mongolia	10 405	696	11 101
Fujian	12 125	477	12 602	Ningxia	1846	427	2272
Gansu	3107	418	3525	Qinghai	953	303	1256
Guangdong	27 402	981	28 383	Shandong	13 546	1445	14 991
Guangxi	10 921	142	11 063	Shanxi	9143	367	9510
Guizhou	10 948	191	11 138	Shaanxi	9585	603	10 187
Hainan	3227	411	3638	Shanghai	4605	141	4746
Hebei	8992	1054	10 046	Sichuan	15 749	330	16 079
Henan	12 614	523	13 137	Tianjin	1392	872	2264
Heilongjiang	10 872	753	11 625	Tibet	769	54	823
Hubei	9278	306	9584	Xinjiang	10 074	1279	11 353
Hunan	9637	465	10 102	Yunnan	8041	208	8249
Jilin	6453	455	6909	Zhejiang	18 790	1305	20 095
Jiangsu	24 272	588	24 859	Average	9393	553	9946
Jiangxi	5786	485	6271	Median	9278	465	10 046
Liaoning	8161	1007	9168	Sum	291 197	17 140	308 337

### C. Intensity of the Epidemic's Impact

**Fig. 5.**
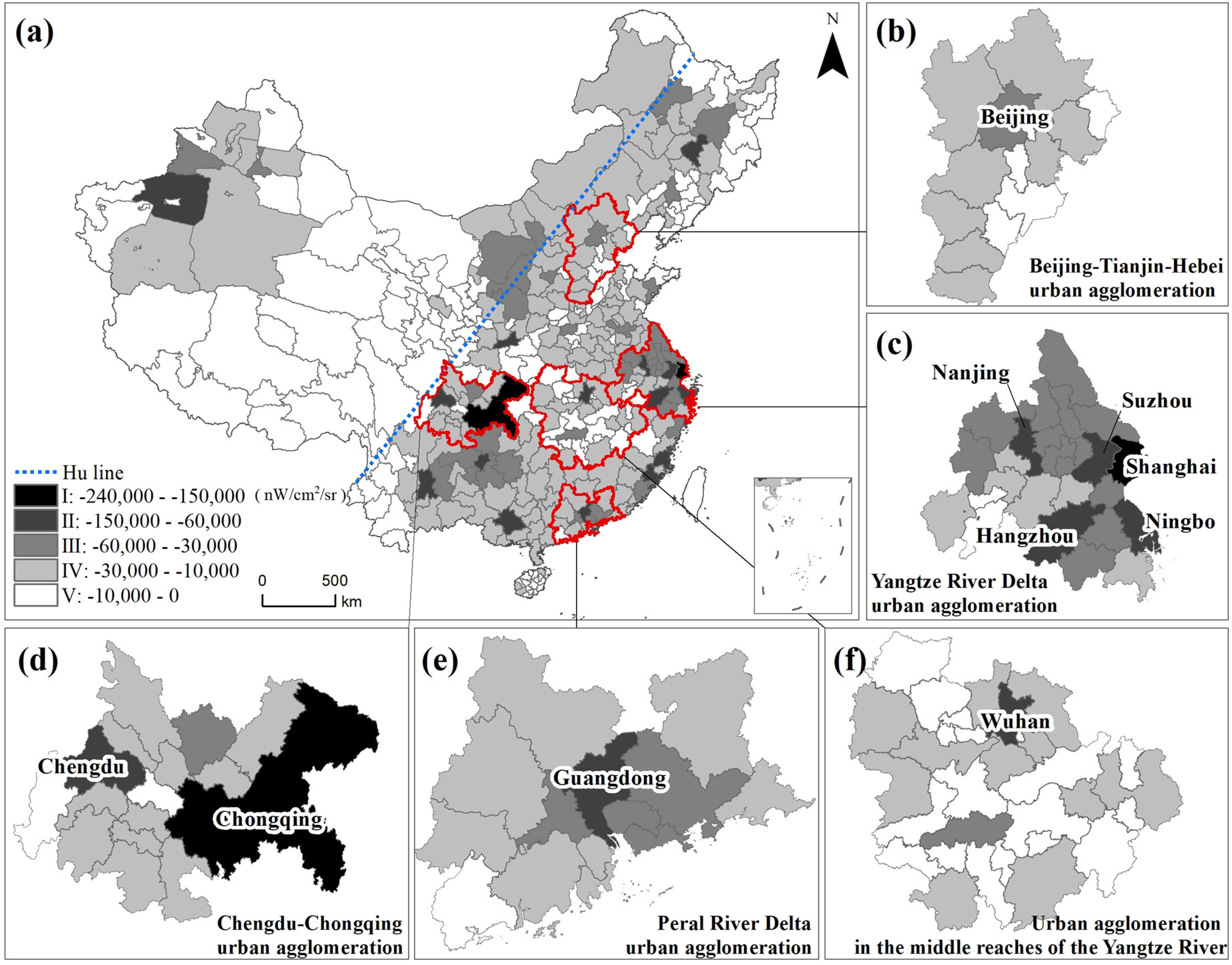
Intensity of the impact of COVID-19 in different urban agglomerations in China.

In this study, we used the natural breaks method to round the influence intensity, and divided the intensity of the influence of COVID-19 into five levels, namely, Ⅰ: −240 000 to −150 000 nW/cm^2^/sr, including two cities (Shanghai and Chongqing); Ⅱ: −150 000 to −60 000 nW/cm^2^/sr, including 15 cities; Ⅲ: −60 000 to −30 000 nW/cm^2^/sr, including 42 cities; Ⅳ: −30 000 to −10 000 nW/cm^2^/sr, including 154 cities; and Ⅴ: −10 000 to 0 nW/cm^2^/sr, including 153 cities (see Fig. 5).

**Fig. 6.**
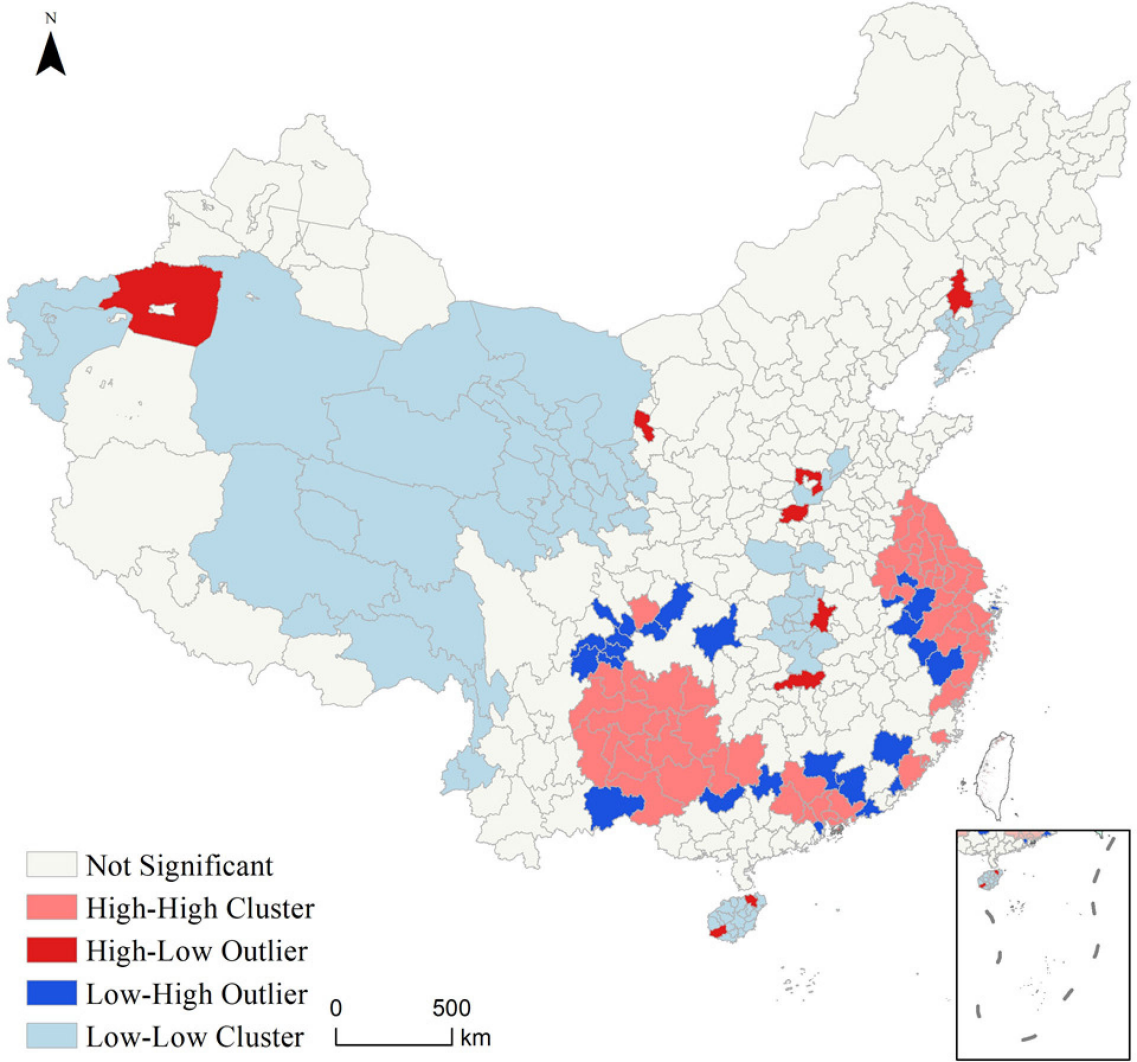
Spatial correlations between cities with different influence intensities.

From a national perspective, the areas most affected by COVID-19 were mainly distributed in the area east of the Hu line and a few cities in West China, followed by a few cities in East, Central, Northeast, and West China. Four categories, namely, high–high clusters (clusters of high influence intensity), high–low outliers (outlier in which a high influence intensity is surrounded primarily by low influence intensity), low–high outliers (outliers in which a low influence intensity is surrounded primarily by high influence intensity), and low–low clusters (cluster of low influence intensity), were defined based on the Anselin Local Moran's I. It was found that the high–high clusters were distributed in coastal areas in Eastern China and some cities in the provinces of Yunnan, Guizhou, and Sichuan, and the Guangxi Zhuang Autonomous Region. Cities with high–low outliers were relatively scattered and included Wuhan, Changsha, and Zhengzhou in Central China, Anyang, Haikou, and Ledong in East China, Shenyang in Northeast China, and Aksu and Yinchuan in West China. The low–high outliers were mainly distributed around the high–high cluster (see Fig. 6).

Among the five major urban agglomerations, the Yangtze River Delta and Chengdu–Chongqing urban agglomerations were the most affected by the epidemic, followed by the middle reaches of the Yangtze River, the Pearl River Delta urban agglomeration, and the Beijing–Tianjin–Hebei urban agglomeration. Shanghai, in the Yangtze River Delta urban agglomeration, was the most affected, at level Ⅰ; in the Chengdu–Chongqing urban agglomerations, Chongqing and Chengdu were the most affected, at levels Ⅰ and Ⅱ, respectively. Wuhan, in the urban agglomerations in the middle reaches of the Yangtze River, was the most affected, at level Ⅱ; Beijing, in the Beijing–Tianjin–Hebei urban agglomeration, was the most affected, at level Ⅲ. The core cities (political centers or economic centers) of each urban agglomeration were often affected to a greater extent (see Fig. 5).

**Fig. 7.**
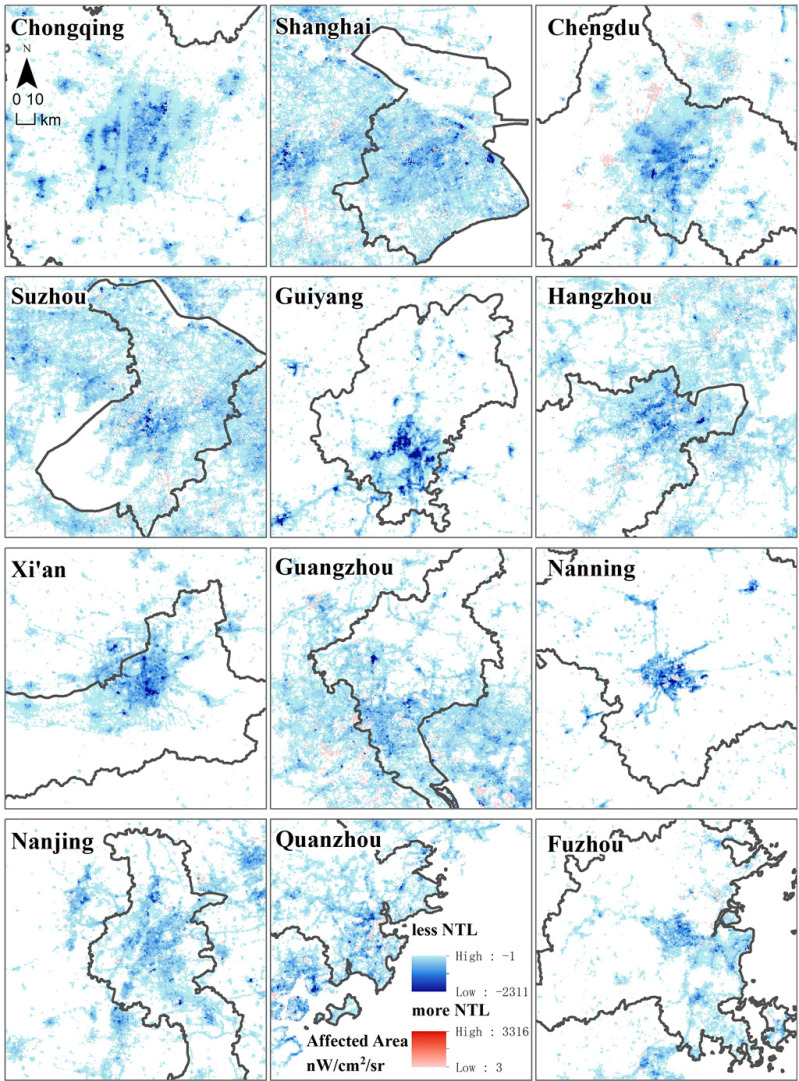
Twelve cities in China most affected by the COVID-19 epidemic.

The 12 most affected cities in China were Chongqing, Shanghai, Chengdu, Suzhou, Guiyang, Hangzhou, Xi'an, Guangzhou, Nanning, Nanjing, Quanzhou, and Fuzhou. These cities are municipalities under the direct administration of the central government or else, are provincial capitals or economic centers. The areas with the greatest impact intensity of COVID-19 in each city were mainly located in the central business district (CBD) of the city, and gradually weakened traveling outwards (see Fig. 7).

### D. Degree of Urban Recovery

**Fig. 8.**
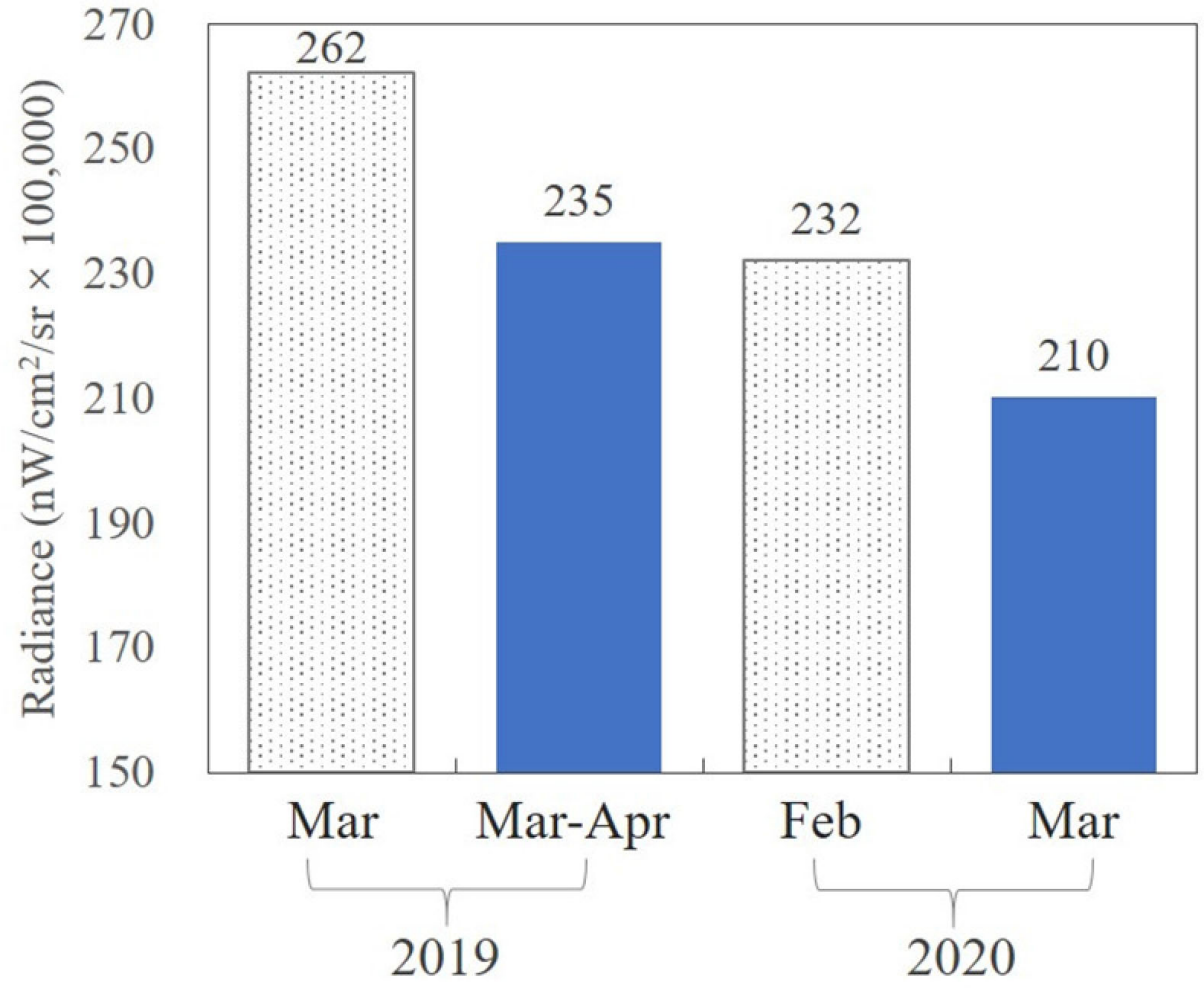
Changes in the total amount of NTL in China in 2019 and 2020 after the Spring Festival. "Feb 2020" refers to 19–29 February 2020 and "Mar 2020" refers to 1–31 March 2020; "Mar 2019" refers to 3–12 March 2019 and "Mar-Apr 2019" refers to 13–31 March 2019 and 1–12 April 2019.

The recovery period was divided into the February recovery period (19–29 February 2020) and the March recovery period (1–31 March 2020) according to the month. In China, the changing trend of the total NTL radiance after the Spring Festival in 2020 was consistent with the changing trend of the total NTL radiance after the Spring Festival in 2019; that was, the total amount of NTL radiance in February 2020 and March 2019 was higher than the total amount of NTL radiance in March 2020 and April 2019. Additionally, the total amount of NTL radiance in 2020 was lower compared to 2019. Generally, in the February recovery period, the overall NTL intensity across China had recovered to 88.53% of the level in the same period in the previous year, while in the March recovery period, the overall NTL intensity across China had recovered to 89.48% of the level in the same period in the previous year (see Fig. 8).

**Fig. 9.**
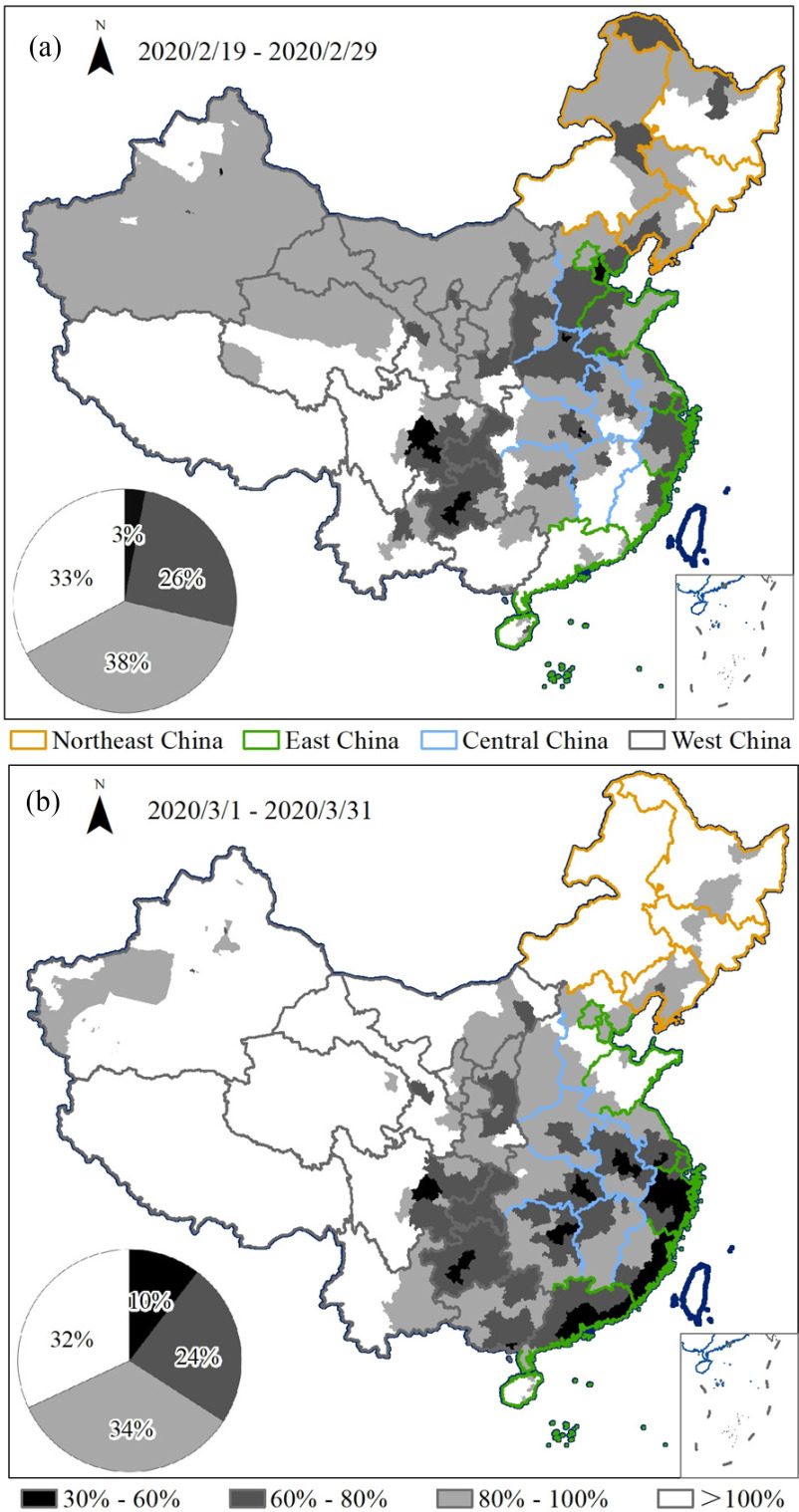
Recovery degree of cities. (a) February recovery period (19–29 February 2020). (b) March recovery period (1–31 March 2020).

City-scale analysis showed that, in all cities, by 31 March 2020, the NTL radiance had returned to over 30% of the level in the same period in the previous year. In the recovery period in February, fully restored cities accounted for 33% of the total, cities with a recovery degree of 80%–100% accounted for 38% of the total, and only 3% of the cities had a recovery degree of 30%–60%. The cities with a recovery degree of 30%–60% were mainly distributed in the Chengdu–Chongqing urban agglomeration, as well as in the Yangtze River Delta urban agglomeration and the Beijing–Tianjin–Hebei urban agglomeration. The fully restored areas included Tibet, Yunnan, and Jilin [see Fig. 9(a)].

By the end of the recovery period in March, compared with the February recovery period, the number of cities with a recovery degree of over 60% had decreased to 90%, while cities with a recovery degree of 30%–60% had increased to 10% of the total. By the end of the recovery period in March, most cities in West and Northeast China had fully recovered, some even exceeding the NTL level at the same point in the Chinese lunar calendar in 2019. However, the recovery degree of the coastal cities in Eastern China was lower compared with the February recovery period, which may be due to the increase in the number of imported COVID-19 cases during this period. These imported cases were mainly distributed in coastal cities, where epidemic prevention and control measures had since been further strengthened and human activities had decreased again, and the NTL radiance of these cities had reduced by the end of the March recovery period. The overall recovery of the Beijing–Tianjin–Hebei urban agglomeration was strong. However, the Chengdu–Chongqing urban agglomeration, the Yangtze River Delta urban agglomeration, the Pearl River Delta urban agglomeration, and the middle Yangtze urban agglomeration had a weaker recovery [see Fig. 9(b)].

**Fig. 10.**
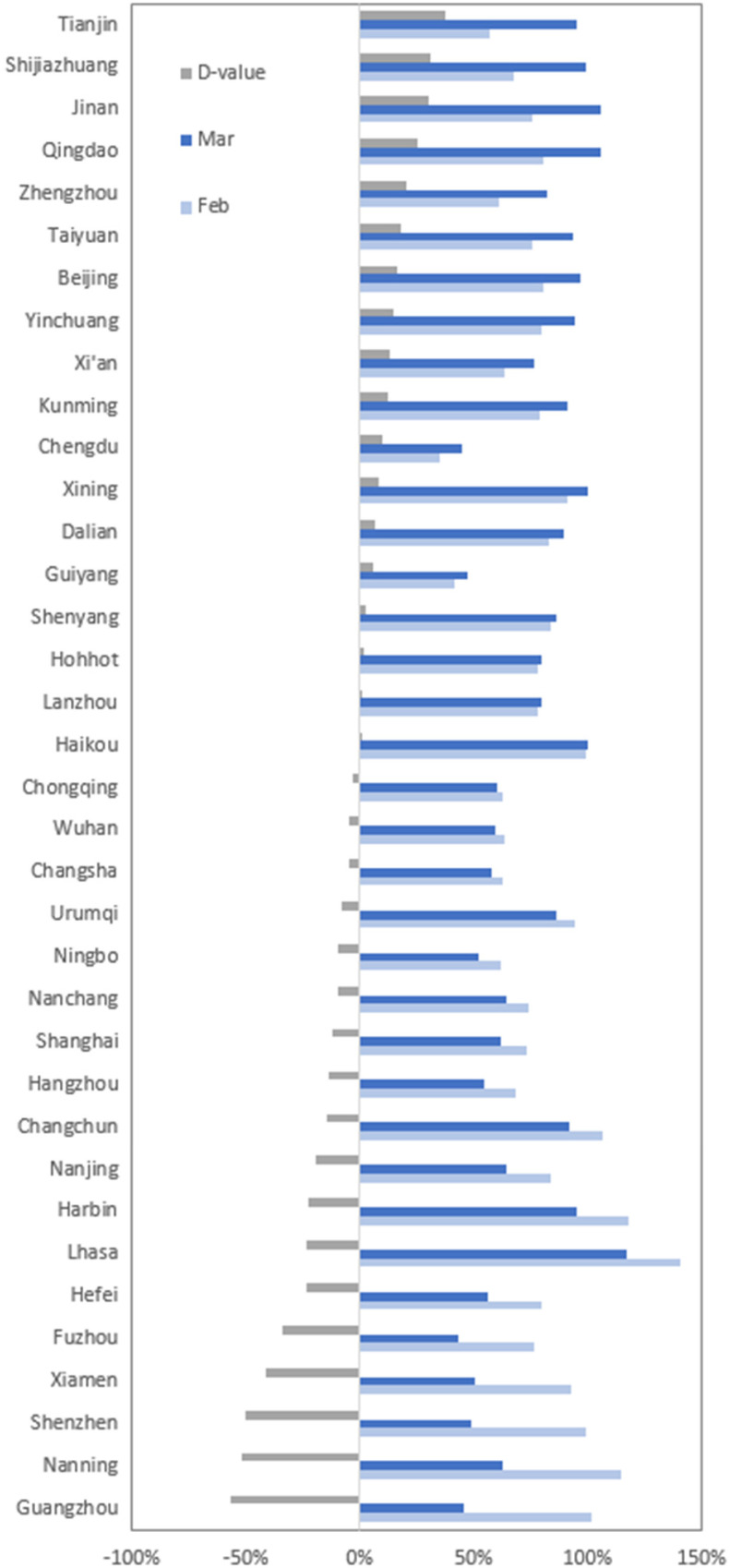
NTL recovery degree of provincial capitals, province-level municipalities, and vice-provincial cities of 31 provincial administrative divisions. The D-value is the difference in recovery degree between the March and February recovery periods.

Fig. 10 shows the NTL recovery degree of provincial capitals, province-level municipalities, and vice-provincial cities—a total of 36 cities—in 31 provincial administrative divisions. In 31 out of the 36 cities, the recovery degree reached more than 50% in both the February and March recovery periods. A total of 50% of cities recovered steadily, with the degree of recovery in March being greater than that in February, whereas the recovery in the remaining 50% of cities showed a certain fluctuation. The recovery degree of the coastal cities of Guangzhou, Nanning, Shenzhen, and Xiamen was 40% lower in March than in February.

Furthermore, IICT was applied to verify the accuracy of cities’ recovery degree. The recovery degree of cities in the February recovery period based on IICT was compared with the recovery degree in the same period based on NTL. It was found that, in the February recovery period, there were 354 cities with a recovery degree of more than 60%, and 300 of these 354 cities had a IICT recovery of more than 60% during the same period. There is a high agreement between the recovery degree determined based on IICT and the recovery degree determined based on NTL, as shown in Table Ⅱ. The trends of the two are similar but not completely consistent. This is reasonable because they are the different dimensions of urban restoration; NTL reflects the urban vitality in space, while IICT is the statistical value of population flow within cities.

**TABLE II:** Number of Cities With Different Recovery Degrees

Recovery degree	NTL	NTL&IICT	IICT
>40%	364	342	343
>50%	362	329	330
>60%	354	300	309
>70%	313	328	273
>80%	260	149	192

### E. Dynamics of NTL in the Center of the Epidemic Outbreak in China

**Fig. 11.**
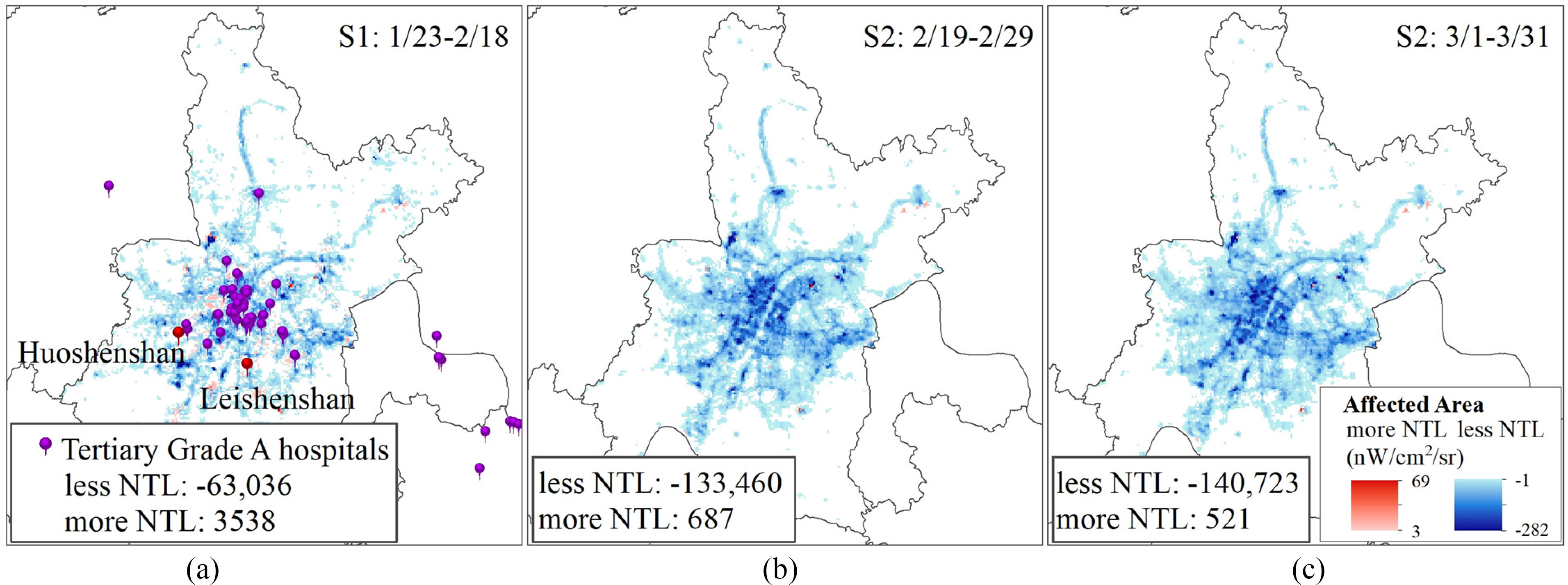
Dynamics of NTL in Wuhan, the center of the epidemic outbreak in China.

During the outbreak period, Wuhan, as the hardest hit city, experienced a significant increase in NTL radiance in the core areas on both sides of the Yangtze River and in the locations of Tertiary Grade A hospitals. After the epidemic reached the turning point, the NTL radiance in Wuhan, including Huoshenshan hospital and Leishenshan hospital, decreased gradually until the end of March (see Fig. 11). During the February recovery period, the total value of NTL radiance in Wuhan was 64% of that in the same period in 2019, and in the March recovery period, it was 60% of that in the same period in 2019. Between February and March 2020, the area with a higher NTL radiance than the same period in 2019 gradually reduced and the area with a lower NTL radiance gradually increased; this reflects the gradual reduction of the prevention and control measures and the effective control of the epidemic.

## V. Discussion

### A. Relationship Between Epidemics and NTL

NTL is closely related to human activities [29]; however, its relationship to epidemics is not yet clear. Therefore, in the context of the COVID-19 outbreak, this study explores the impact of the outbreak on NTL in China using high-temporal-resolution NTL remote sensing data. First, we found that the severity of the epidemic was not the direct cause of the change of NTL (the biggest R^2^ value of the linear regression between the number of COVID-19 cases and NTL changes at different times is only 0.098). After the outbreak, the prevention and control measures caused changes in human activities, which in turn caused changes in NTL. This is different from the NTL changes caused by war and natural disasters (e.g., typhoons, earthquakes, and flooding), which are caused by damage to power supply systems and other infrastructure, which directly changes NTL. The NTL is normal before the war or natural disaster, the NTL is dimmed during the process, and the NTL gradually brightens during the recovery period [22], [23]. However, for epidemics, the NTL changes have nothing to do with damage to infrastructure but are mainly caused by changes in human activities. For example, Wuhan, the center of the COVID-19 outbreak in mainland China, had an area of 2197 km^2^ in which the NTL was reduced during the epidemic, ranking 22nd among 368 cities in China; the total reduction in NTL intensity was −63 061 nW/cm^2^/sr, ranking 16th; and the total increase in NTL intensity was 3542 nW/cm^2^/sr, ranking 39th. This suggests that neither the area of NTL reduction nor the magnitude of the intensity change can be used to determine the severity of the epidemic.

However, the overall change trend of NTL can indirectly reflect the occurrence of an epidemic in a city, as well as the approximate severity of the epidemic. Epidemics indirectly cause changes in NTL by affecting human activities; that is, changes in human activities in response to the epidemic will directly cause changes in NTL. Under the influence of COVID-19, the NTL of all cities in China has been reduced to varying degrees, and cities which experienced a greater reduction in NTL are concentrated to the east of the Hu line, and these cities are also severely affected by COVID-19. Tibet, which had the lowest number of COVID-19 cases of any region of China, had the lowest change in NTL and the best recovery. Therefore, these results suggest that changes in NTL can be used to make a general judgment about the severity of outbreaks in cities.

### B. Significance of NTL Monitoring

**Fig. 12.**
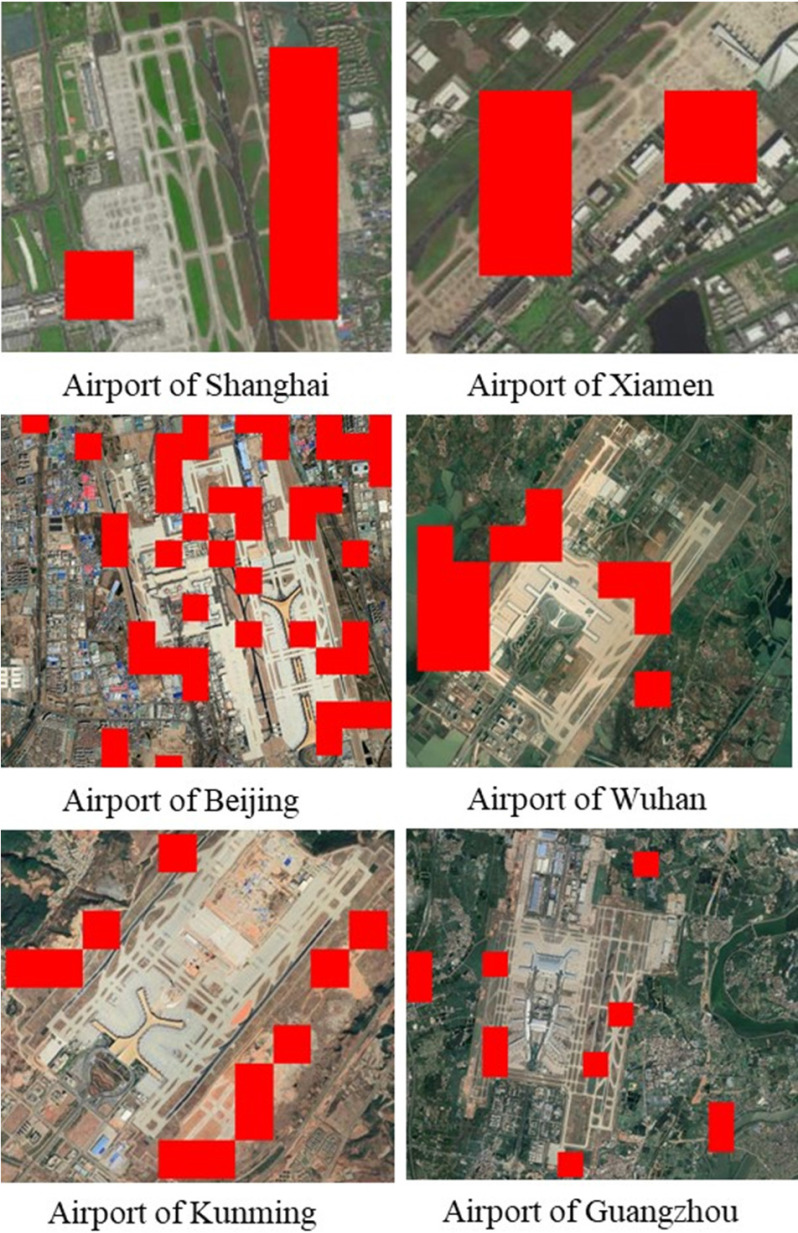
Representations of the increase of NTL at airports during the COVID-19 epidemic. Red areas indicate areas where NTL increased in February 2020 compared to the same period in 2019; the pixel size is 500 × 500 m.

Under the influence of COVID-19, the affected areas include areas where the NTL radiance becomes lower, and areas where the NTL radiance becomes higher. The decrease of NTL over large areas indicates the weakening or cessation of social and economic activities, while the increase of NTL in a few areas was caused by epidemic prevention and control measures or the increased use of infrastructure such as hospitals. For example, during the outbreak period, although the number of daily flights at airports decreased, the NTL of many airports was brighter than before the outbreak of COVID-19 due to an increase in the brightness of high pole lights in order to facilitate epidemic prevention and control measures (see Fig. 12). Moreover, the NTL at the entrances and exits of some expressways also increased during the epidemic due to the implementation of active monitoring and control measures for COVID-19. Therefore, the monitoring of NTL changes in specific locations can be used to evaluate the effectiveness of urban prevention and control measures for the epidemic.

**Fig. 13.**
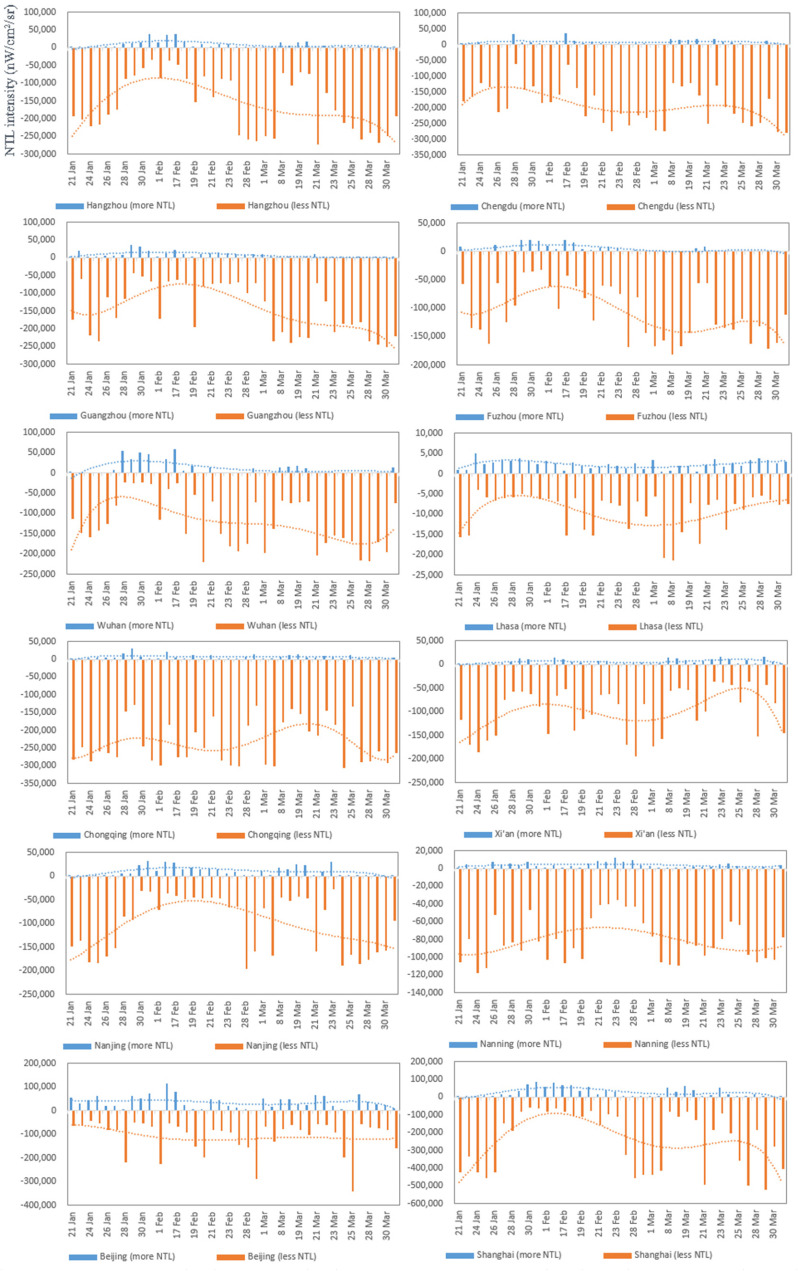
Changes in daily NTL intensity compared to the same period in the previous year in 12 cities in China during the COVID-19 outbreak. The I-line is the NTL change trend line in the areas where the NTL intensity in January–March 2020 was higher than that during the same period in 2019; the D-line is the NTL change trend line in the areas where the NTL intensity in January–March 2020 was lower than that during the same period in 2019.

The change trends in the daily NTL intensity can also be used to reflect the impact of the epidemic in a city as well as the city's recovery process. Fig. 13 shows the changes in daily NTL intensity in 12 cities in China during the COVID-19 outbreak. The NTL change trend lines, including trend lines in the areas where the NTL intensity in January–March 2020 was lower than that in the same period in 2019 (hereinafter called D-lines) and trend lines in the areas where the NTL intensity in January–March 2020 was higher than that in the same period in 2019 (hereinafter called I-lines), were obtained by fitting a fifth-order polynomial to the change in the daily NTL intensity. Then, according to the D-line, cities are divided into two types, namely, M-type and inverted-U-type; the M-type cities have two recovery periods and the inverted-U-type cities have one recovery period. When the tail of the D-line is upward, it indicates that a good recovery will occur in the future (e.g., Lhasa, Nanning, Chongqing); when the tail of the D-line is downward, it indicates a recurrence of the epidemic (e.g., Fuzhou, Guangzhou). Additionally, the I-line can show the intensity of urban epidemic prevention and control measures.

### C. Characteristics of the Affected Cities

These affected areas have similar characteristics at the national, provincial, and urban scales—at the national scale, the economic center Shanghai is the most affected, at level Ⅰ; at the provincial scale, the economic and political centers of the provinces are more affected; at the city scale, the central areas of cities are more affected. The cities that were most affected by the outbreak of COVID-19 mostly have fast-growing economies and a high demand for labor, which causes these cities to have a huge “floating population” of migrant workers. Therefore, important economic or political centers of a country should particularly focus on controls during the COVID-19 epidemic to ensure their rapid and safe recovery.

### D. Limitations and Prospects

During the processing of NPP-VIIRS DNB daily data, in order to quickly and accurately eliminate the impact of moonlight, this study directly removed the data affected by moonlight; accordingly, only 15 days of data per month could be used for analysis, thus reducing the temporal resolution of the analysis. In the future, we will explore ways to quickly eliminate the effects of moonlight. Besides, due to time constraints and other reasons, we only analyzed the NTL changes in the severe period of the COVID-19 outbreak (23 January–31 March 2020). In the future, we will investigate the changes in the impact of the COVID-19 outbreak in the period after 31 March 2020.

## VI. Conclusion

The outbreak of COVID-19 before and after the Chinese Spring Festival in 2020 caused drastic changes in human activities and NTL as a consequence. Under this circumstance, this study takes 31 provincial administrative regions in the mainland of China as the research subject and uses high-temporal-resolution NTL data (NPP-VIIRS DNB daily data) to explore the impact of the epidemic and the degree of work resumption in China. The high agreement between the big data of inner city travel intensity and the NTL data proves the validity of the results in this study. The results show that the application of daily satellite-based NTL data is effective to detect dynamic changes in social and economic activities caused by public health emergencies (e.g., wide-range epidemics) in a timely and effective manner.

During the outbreak, the main provincial capitals and urban agglomerations were relatively more affected, and these locations were mainly distributed to the east of the Hu line. During the outbreak, the NTL intensities of five major urban agglomerations decreased to different degrees. Among them, the Yangtze River Delta urban agglomeration, the Chengdu–Chongqing urban agglomeration, and the Pearl River Delta urban agglomeration of the Guangdong–Hong Kong–Macao Greater Bay Area were most affected. Clusters with H-H impact intensity have a relatively small distribution range and cities with H-L outliers are scattered, indicating that prevention and control measures have played a positive role in hindering the spread of COVID-19. By the end of March 2020, 66% of cities in China had recovered by more than 80% compared to the same period in the previous year. Cities in West and Northeast China recovered well, whereas the degree of recovery in Central and Eastern China was relatively low and showed a certain fluctuation due to an increase in the number of imported cases.

More importantly, we found that the change in NTL intensity is not correlated to the number of cases, but reflects the changes in human activities and the effectiveness of epidemic prevention and control measures. However, the overall change trend of NTL can be an indication to the status of an epidemic in a city. Furthermore, the fitted trend of daily NTL changes can provide an effective measure for monitoring the effectiveness of epidemic prevention and control measures, and can also be used to reflect the spatiotemporal recovery characteristics of each city and predict the future recovery trend in a city.
